# Breast cancer liver metastasis: Pathogenesis and clinical implications

**DOI:** 10.3389/fonc.2022.1043771

**Published:** 2022-10-25

**Authors:** Cuiwei Liu, Srivarshini C. Mohan, Jielin Wei, Ekihiro Seki, Manran Liu, Reva Basho, Armando E. Giuliano, Yanxia Zhao, Xiaojiang Cui

**Affiliations:** ^1^ Cancer Center, Union Hospital, Tongji Medical College, Huazhong University of Science and Technology, Wuhan, China; ^2^ Department of Surgery, Samuel Oschin Comprehensive Cancer Institute, Cedars-Sinai Medical Center, Los Angeles, CA, United States; ^3^ Department of Biomedical Sciences, Samuel Oschin Comprehensive Cancer Institute, Cedars-Sinai Medical Center, Los Angeles, CA, United States; ^4^ Key Laboratory of Laboratory Medical Diagnostics, Chinese Ministry of Education, Chongqing Medical University, Chongqing, China; ^5^ The Lawrence J. Ellison Institute for Transformative Medicine, Los Angeles, CA, United States

**Keywords:** liver metastasis, pathogenesis, clinical implications, breast cancer, hepatic microenvironment

## Abstract

Breast cancer is the most common malignant disease in female patients worldwide and can spread to almost every place in the human body, most frequently metastasizing to lymph nodes, bones, lungs, liver and brain. The liver is a common metastatic location for solid cancers as a whole, and it is also the third most common metastatic site for breast cancer. Breast cancer liver metastasis (BCLM) is a complex process. Although the hepatic microenvironment and liver sinusoidal structure are crucial factors for the initial arrest of breast cancer and progression within the liver, the biological basis of BCLM remains to be elucidated. Importantly, further understanding of the interaction between breast cancer cells and hepatic microenvironment in the liver metastasis of breast cancer will suggest ways for the development of effective therapy and prevention strategies for BCLM. In this review, we provide an overview of the recent advances in the understanding of the molecular mechanisms of the hepatic microenvironment in BCLM formation and discuss current systemic therapies for treating patients with BCLM as well as potential therapeutic development based on the liver microenvironment-associated signaling proteins governing BCLM.

## Introduction

Breast cancer (BC) is the most common malignant disease in female patients worldwide (1, 2). Intrinsic BC subtypes by gene expression profiling include luminal A, luminal B, luminal/human epithelial growth factor receptor 2 (HER-2), HER-2 enriched, basal-like, and triple-negative (TN) non-basal (3). Currently, the 5-year survival rate for BC is over 90%. However, about 50% of patients diagnosed with BC will develop distant metastases (4), and the 5-year survival rate declines to less than 20% once distant metastases have developed (5, 6).

BC can spread to almost every place in human body, most frequently metastasizing to lymph nodes, bones, lungs, liver, and brain (7–9). The liver is one of the most common metastatic locations for solid malignant tumors, and it is also the third common metastatic organ for BC (10). Patients with breast cancer liver metastasis (BCLM) often suffer deterioration of liver function due to the aggravation of BC burden, which will threaten the lives of BC patients (11). The survival is only 4-8 months if BCLM is left untreated (12). Therefore, the treatment of BCLM is a significant issue globally. Thus far, no standard therapy has been established for BCLM (13). Currently, the treatments for BCLM include chemotherapy, immunotherapy (triple negative disease), targeted systemic therapies including endocrine therapy (luminal subtype), HER-2 target therapy (HER-2 enriched subtype), radiotherapy, and palliative therapy (11, 14). However, patients with BCLM frequently exhibit poor response to the current therapies and experience high mortality rates (15).

BCLM is a complex process. Its biological basis has not been well delineated. It has been found that the hepatic microenvironment plays a significant role in BCLM (16). An understanding of hepatic microenvironment in the liver colonization of metastatic BC cells is essential for developing novel and effective therapy for BCLM. In this review, we provide an overview of recent advances in molecular mechanisms of the hepatic microenvironment in BCLM formation and discuss current systemic therapies as well as potential therapeutic development based on the liver microenvironment-associated signaling proteins governing BCLM.

## Organ tropism of breast cancer metastasis

It is a long-standing observation that different subtypes of BC show distinct propensity of metastasizing to specific organs (3, 17, 18). Luminal breast cancer (LBC) preferentially metastasizes to the bones, while HER-2 and basal-like BC often develop visceral metastases including brain, liver and lung metastasis (19). Some studies report that the HER-2 enriched subtype found to exhibit a higher risk of developing liver metastasis (18, 20, 21). In contrast, other studies report that basal-like BC has a lower rate of liver metastasis (3). Although there are some discrepancies in reports about preferential organ sites of breast cancer metastasis, it is now accepted that particular metastatic sites are associated with different breast cancer subtypes (22).

It is well-established that preferred metastatic sites are mechanistically determined by molecular, cellular and microenvironment factors rather than random dissemination (23). Features of organ circulation may have a key role in determining the sites of metastatic disease as capillary networks in tissue arrest the circulating BC cells (10). Organs that receive similar amounts of blood and circulating tumor cells show differing abilities to accommodate disseminating BC cells and form metastases. This finding indicates that the “mechanical arrest” may not be the only explanation for organ tropism of BC metastasis (24, 25). Another explanation is the “seed and soil” hypothesis, which proposes the metastases form only when the disseminated BC cells are compatible to the distant organ microenvironment (26). The ability of BC cells to interact with tissue resident cells and the microenvironment factors may also determine the metastatic organs of BC (24). Therefore, the crosstalk between BC cells and liver tissue components provides key mechanisms that dictate BCLM (27–29). In this review, we will describe and summarize our current knowledge on the BCLM process.

## Metastatic phase of breast cancer liver metastasis

**Figure 1:**
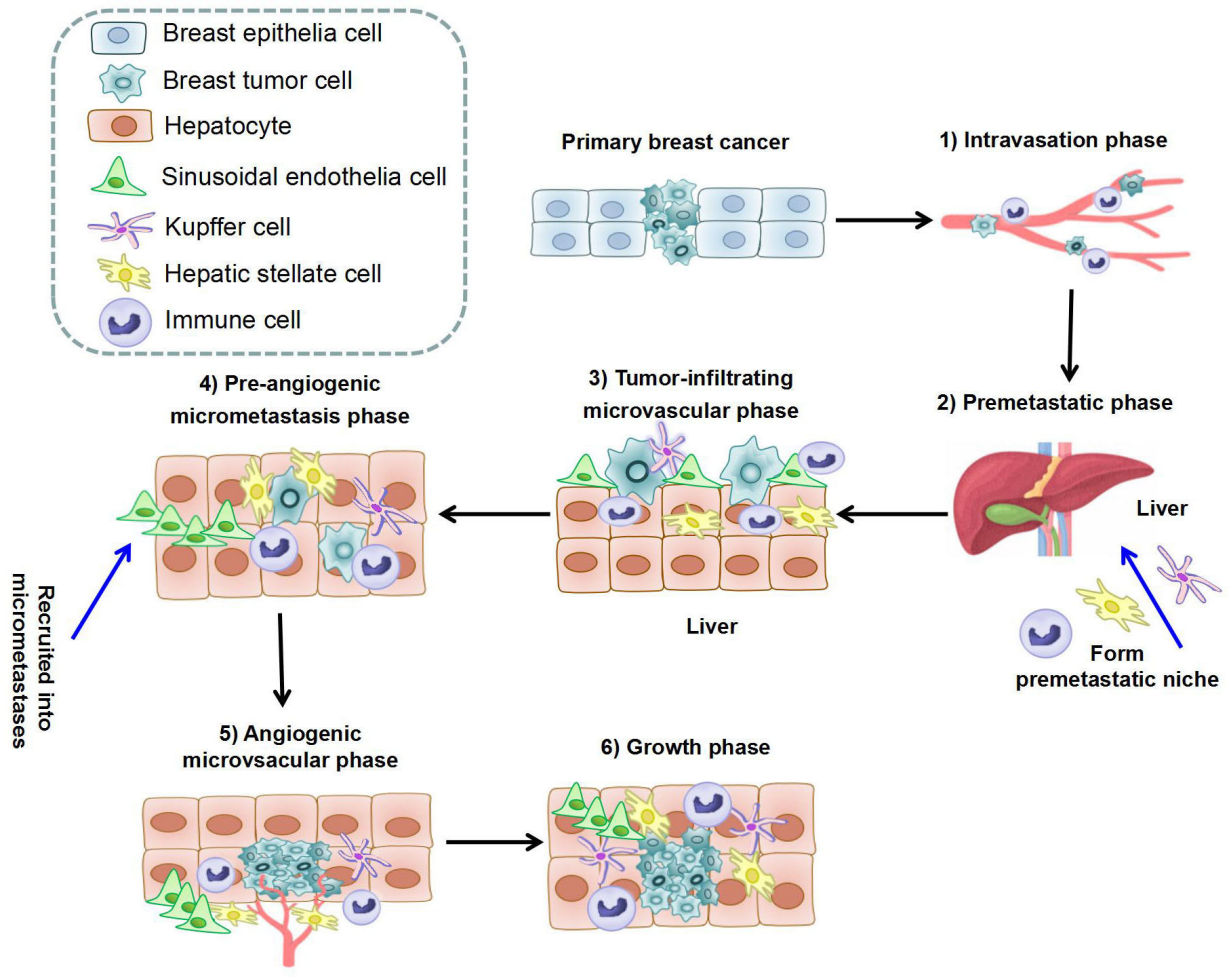
The process of liver metastasis formation in breast cancer. The whole process can be separated into six steps: 1) Intravasation phase: cancer cells intravasate into the circulation system under help of immune cells; 2) Premetastatic phase: HSCs, KCs, and immune cells in hepatic microenvironment form premetastatic niche; 3) Tumor-infiltrating microvascular phase: cancer cells extravasate into liver parenchyma through LSECs; 4) Pre-angiogenic micrometastatic phase: HSCs and immune cells are recruited into micrometastases and activate local stromal response; 5) Angiogenic micrometastatic phase: micrometastases become vascularized and interact with cells in the microenvironment; 6) Growth phase: metastases expansion under the stimulation of hepatocytes, HSCs, and immune cells. (HSC, Hepatic stellate cell; KC, Kupffer cell; LSEC, Liver sinusoidal endothelial cells).

The formation of BCLM involves a series of complex biological processes. The BC cells will undergo epithelial-to-mesenchymal transition (EMT), detach from the primary tumor, and intravasate through endothelial barriers into the blood circulation system (30, 31). Macrophages and mesenchymal stem cells (MSCs) contribute to EMT at primary BC. Cancer-associated fibroblasts (CAFs) and myeloid progenitor cells are recruited to invasive edge of primary BC and promote intravasation (31). Platelets are also involved in the survival of BC cells in circulation to extravasation sites (31). After circulating tumor cells extravasate into the parenchyma of the liver (32),they will enter a dormant state or form clinically detectable macrometastases (33, 34). BCLM is considered to comprise multi-steps: 1) The intravasation phase; 2) The premetastatic phase (35–37); 3) the tumor-infiltrating microvascular phase; 4) the pre-angiogenic micrometastatic phase; 5) the angiogenic micrometastatic phase; 6) the growth phase (38). The process of BCLM is summarized in **Table 1** and **Figure 1**.

**Table 1:** The phases of breast cancer liver metastasis.

Phase of liver metastasis	Function	Involved cells	References
1. The intravasation phase	Primary breast cancer cells detach from surrounding cells and intravasate into the circulation system	MSCs; macrophages; endothelial cells; platelets; CAFs; myeloid progenitor cells; DCs; neutrophils	(30, 31, 39, 40)
2. The premetastatic phase	Form “premetastatic niche” in the liver permit breast cancer cells entry and outgrowth	HSCs; CAFs; KCs; MDSCs; Tregs; neutrophils	(35–37, 41–48)
3. The tumor-infiltrating microvascular phase	Breast cancer cells arrest in the sinusoidal vessels and lead to cancer cell extravasation	M2 macrophages; HSCs; CAFs; neutrophils; LSECs; hepatocytes; KCs	(32–34, 39, 40, 49–56)
4. The pre-angiogenic micrometastasis phase	Host stromal cells are recruited into avascular micrometastases in the liver	M2 macrophages; LSECs; neutrophils; HSCs; KCs	(40, 53, 56–63)
5. The angiogenic micrometastasis phase	Metastatic breast tumors in the liver become vascularized through several possible interactions with the microenvironment	M2 macrophages; LSECs; neutrophils; HSCs; KCs	(40, 53, 56–63)
6. The growth phase	Metastatic breast tumors in the liver become a “clinical” macrometastases	Hepatocyes; HSCs; LSECs; M2 macrophages; neutrophils; MDSCs; Tregs; KCs	(38, 47, 48, 56–61, 64, 65)

MSC, Mesenchymal stem cells; DC, Dendritic cell; HSC, Hepatic stellate cell; CAF, Cancer-associated Fibroblast; KC, Kupffer cell; MDSC, Myeloid-derived suppressor cell; Treg, Regulatory T cell; LSEC, Liver sinusoidal endothelial cells.

Of the aforementioned steps, the “premetastatic phase” is essential for organ-specific metastasis formation and has recently gained much attention. Numerous studies have proposed that primary tumor-derived secreting factors are associated with the premetastatic niche formation in distant organs (36, 66). The vascular endothlial growth factor (VEGF) and transforming growth factor-β (TGF-β) show critical roles in premetastatic niche formation to promote BC metastasis (41–43). Moreover, chronic psychological stress can promote metastatic colonization of circulating BC cells by promoting a premetastatic niche through activating β-adrenergic signaling (36). BC secreted exosomes can fuse preferentially with organ-specific cells at their predicted destination to prepare the premetastatic niche and exosomal integrins can be used to predict organ-specific metastasis (44). In the liver, exosomes secreted from BC cells can reach liver and fuse with Kupffer cells to form premetastatic niche (44). Exosomal integrin αvβ5 uptake by Kupffer cells can induce Src phosphorylation and *S100* gene expression to determine liver metastasis (44). Tumor-derived tissue inhibitor of metalloproteinases 1 (TIMP-1) was also found to induce liver metastasis *via* hepatic stromal cell derived factor 1 (SDF-1) and neutrophil recruitment (45). Despite these recent discoveries, the roles and mechanisms of premetastatic step in BCLM are currently not well understood.

## Hepatic microenvironment of breast cancer liver metastasis

The hepatic microenvironment into which disseminated BC cells invade and colonize is pivotal for the formation of BCLM. The hepatic microenvironment is highly regulated, relying heavily on the interaction between BC cells and resident cell populations (67). These interactions help nurture liver tissue to become fertile grounds for tumor cell seeding. The roles of different types of cells in the microenvironment of liver metastases are summarized in **Table 2** and described below.

**Table 2:** The roles of different cells in the microenvironment of breast cancer liver metastasis.

Cell type	Molecules or cytokines	Interaction with other cells
Cancer stem cells (CSCs)	TGF-β1 pathway promote liver metastasis of breast cancer by inducing the CD44^high^/CD24^-^ breast cancer stem cell population (68, 69)	Interaction between CSCs and liver microenvironment cells promote metastasis (70, 71)
Liver sinusoidal endothelial cells (LSECs)	TNF-α or IL-1 stimulate the attachment of tumor cells to LSECs and lead extravasation (49–51); LSECs secret fibronectin induce EMT and promote metastasis (72); CXCL12, ICAM-1, STAT3, PD-L1 and microRNA-20a expressed by LSECs interact with cancer cells and involve in liver metastasis (64)	Obstruction of the sinusoids by tumor cells can lead ischemia, trigger inflammatory response and damage disseminated tumor cells (73–78)
Hepatocytes	Claudin-2 (52, 79–81), E-cadherin (82) promote the adhesion between tumor cells and hepatocytes; hepatocytes release IGF-1 and HGF to promote metastasis (53); HGF-like protein secreted by hepatocytes activate RON to promote metastasis (53)	Tumor-hepatocyte interactions promote liver metastasis (52, 79–83)
Liver macrophages	M1 to M2 repolarization induced by IL-4, IL-13 and STAT6 pathway contribute to metastasis (57, 58, 84, 85); PLD-2 promote TAMs infiltration in breast tumor and liver metastasis (86)	M2 macrophage phenotype regulate EMT of breast cancer cells, and promote liver metastasis (39)
Kupffer cells (KCs)	KCs relase oxygen metabolites, cytotoxic cytokines, proteases, TNF-α and IL-1β to damage disseminated tumor cells (53, 85, 87, 88); KCs decrease cancer cells by promoting secreting GM-CSF and IFN-γ (53); KCs release growth factors (HGF, VEGF), cytokines (TNF-α, Il-1, IL-1β, IL-6 and IL-10), MMP9 and MMP14 to promote extravasation (49–51, 53) and outgrowth of metastases (53, 59–61)	KCs fused with exosomes secreted from cancer cells and contributed to the premetastatic niche formation (44); KCs damage disseminated tumor cells through recruitment of NK cells (85, 87, 88)
Cancer-associated Fibroblasts (CAFs)	CAFs promote metastasis through exhibiting antitumor immune suppression depends on CXCL12 or NOX4 signaling (89, 90)	CAFs modify ECM which may facilitate cancer cell migration or act as barrier (91)
Hepatic stellate cells (HSCs)	Activated HSCs promote metastasis through exhibiting antitumor immune suppression response by releasing potent immune suppressor TGF-β (46); HIF-1 activates TWIST and promotes the binding of VEGF to VEGFR to contribute liver metastasis (92); RLN target activated HSCs inhibit metastasis (92)	Activated HSCs promote metastasis by organization of ECs into neo-vessel network (62) and inducing LSECs and ECs to form vascular tube (63); activated HSCs promote metastasis by inducing T cell apoptosis and NK cells quiescence (93, 94); HSCs modify ECM which may facilitate cancer cell migration or act as barrier (91, 95)
Neutrophils	Neutrophils inhibit tumor growth by releasing cytolytic factors (40); aged neutrophil promote metastsis by releasing promoting factors (96); neutrophil-derived transferrin promote metastasis (97); loss of p53 in cancer cells triggers WNT-dependent systemic inflammation promote metastasis (98)	Neutrophils inhibit tumor growth through recruiting CD8^+^ cytotoxic T cells or macrophages (54, 99); physical interaction of neutrophils with tumor cells enhance migration of tumor cells into the extravascular space (55)
Myeloid-derived suppressor cells (MDSCs)	MDSCs can be recruited to the metastases by chemokines (CXCL1 and CXCL2) (100); S100A8/Gr1-positive MDSCs (101) can promote growth and aggressiveness of cancer cells by producing arginase and IL-6 (102–104)	Tumor cells recruiting MDSCs can induce immune tolerance state to contribute tumor growth (47, 48)
Regulatory T cells (Tregs)	–	Tregs contribute metastasis by inhibiting antitumorigenic T-cell (65)

TAM, Tumor-associated macrophage; EMT, Epithelial-to-mesenchymal transition; NK cells, Natural killer cells; ECM, Extracellular matrix; EC, Endothelial cell; TGF-β, Transforming growth factor-β; TNF, Tumor necrosis factor; CXCL, Chemokine (C-X-C Motif) ligand; NOX4, Nicotinamide adenine dinucleotide phosphate oxidase 4; IL, Interleukin; STAT, Signal transducer and activator of transcription; PLD, Phospholipase D; HGF, Hepatocyte growth factor; ICAM-1, Intercellular adhesion molecule 1; PD-L1, Programmed cell death-ligand 1; IGF-1, Insulin-like growth factor 1; HIF, Hypoxia induced factor; GM-CSF, Granulocyte macrophage colony stimulating factor; IFN-γ, Interferon γ; MMP, Matrix metalloproteinase; VEGF, Vascular endothlial growth factor; VEGFR, Vascular endothelial growth factor recepter; RLN, Relaxin.

### Cancer stem cells

Cancer stem cells (CSCs) can interact with the hepatic microenvironment such as extracellular matrix, hypoxia or growth factors, all of which contribute to the metastasis (70). Knaack et al. cultured pancreatic cancer CSCs *in vitro* together with hepatic stellate cells and myofibroblasts to demonstrate the importance of these stromal cells in liver metastasis formation (71), indicating a connection between CSCs and liver microenvironment. Furthermore, Zhang et al. showed the CD44^high^/CD24^-^ breast CSC population can activate TGF-β1 signaling and increase the invasive capacity and liver metastasis of BC (68). In line with this study, the cell surface adhesion molecule CD44 was found to enhance breast tumor invasion and metastasis to the liver (69).

### Liver sinusoidal endothelial cells

When breast tumor cells enter the hepatic microcirculation, they first encounter liver sinusoidal endothelial cells (LSECs). LSECs are double-edged swords, as they can not only promote but also inhibit BCLM formation in the hepatic tissue microenvironment.

Regarding the tumoricidal activities of LSECs, many studies have shown that the tumor cells can obstruct the sinusoids to trigger an ischemia and inflammatory response. LSECs release cytotoxic cytokines, which have damaging effects in adjacent tumor cells (73–77). LSECs can also remove or degrade the enzymes that promote angiogenesis and metastasis (78). Whether the tumoricidal action of LSECs exert a prominent effect on BCLM remains to be determined.

On the other hand, tumor cells can activate Kupffer cells (KCs) to secrete proinflammatory cytokines, which induce LSECs to express adhesion molecules and help tumor cells extravasate into hepatic parenchyma (49–51, 72). Also, LSECs allow tumor cells directly adhere to the membrane proteins and promote metastasis (105). Although these findings came from studies on colorectal cancer or lung cancer, it is postulated that LSECs also possess tumor-promoting activities in BCLM.

Furthermore, some critical molecules from LSECs or their surrounding microenvironment are involved in liver metastasis (64). The expression of chemokine (C-X-C motif) receptor 4(CXCR4) in cancer cells is associated with increased expression of chemokine ligand CXCL12 in LSECs’ microenvironment and CXCR4-CXCL12 signaling drives metastasis (64). Intercellular adhesion molecule 1 (ICAM-1), signal transducer and activator of transcription (STAT)3, programmed cell death-ligand 1 (PD-L1), and microRNA-20a with its targeted proteins expressed by LSECs also play a pivotal role in the interaction between LSECs and cancer cells and thereby promote liver metastasis (64).

### Hepatocytes

The main function of hepatocytes is helping metastatic BC cells in seeding and colonization of the liver. BC cells can directly interact with hepatocytes by forming tight-junction-like complexes with hepatocytes in the Disse space, the space between hepatocytes and sinusoids (83). Interestingly, metastatic BC cells exhibit a lower adherent ability with LSECs compared to the hepatocytes (52), suggesting that hepatocytes facilitate BC cell seeding in the liver.

Hepatocytes can release growth factors, such as insulin-like growth factor 1 (IGF-1) and hepatocyte growth factor (HGF) which promote liver metastasis (53). The overexpression of RON receptor has been reported in BC and RON can be activated by HGF-like protein secreted by hepatocytes. RON activation promote cancer cells growth, invasion and metastasis (53).

### Liver macrophages and Kupffer cells

Liver macrophages can be divided into monocyte-derived recruited macrophages and liver resident KCs (84). The M1 to M2 repolarization of tumor-associated macrophages (TAMs) can prevent immunogenic, inflammatory responses while inducing neoangiogenesis and matrix remodeling, thus promoting breast cancer progression and metastasis (57, 58). It has been demonstrated that the EMT of BC cells is regulated by M2 macrophages in the liver metastatic microenvironment (39).

KCs, unlike monocyte-derived recruited macrophages, are permanent resident monocytes in the sinusoids. They can fuse with exosomes derived from BC cells and contribute to the premetastatic niche formation (44). On one hand, KCs can exhibit tumoricidal activity by releasing reactive oxygen species (ROS), cytotoxic cytokines, proteases, and recruitment of other inflammatory cells (85, 87, 88), particularly when the burden of tumor cells invading liver is excessive. The anti-tumor activity of KCs might base on the reruitment of natural killer (NK) cells by secreting inflammatory factors granulocyte macrophage colony stimulating factor (GM-CSF) and interferonγ(IFN-γ) (53). KCs can also decrease metastatic BC by increasing level of tumor necrosis factor (TNF)-α and interleukin (IL)-1β (53). On the other hand, KCs can also promote liver metastasis through secreting growth factors and cytokines including HGF, VEGF, IL-6, matrix metalloproteinase (MMP)9 and MMP14, as demonstrated in colorectal cancer studies (53, 59–61). However, whether KCs have these dichotomous effects in BCLM awaits to be examined.

### Cancer-associated fibroblasts and hepatic stellate cells

The cancer-associated fibroblasts (CAFs) in the hepatic microenvironment are widely considered to be derived from hepatic stellate cells (HSCs) (106). It was found that HSCs can be induced to trans-differentiate into a proliferative and motile form called myofibroblasts by growth factors released from tumor cells or KCs during the development of micrometastases (107, 108). Functionally, activation of HSCs promotes liver metastasis by enhancing tumor cell adhesion, invasion, survival, and proliferation (56). Activated HSCs can also initiate angiogenesis by organizing endothelial cells (ECs) into a neovascular network and inducing LSECs and ECs to form vascular tubes within metastases (62, 63). Vascular endothlial growth factor receptor (VEGFR) are mainly distributed on the endothelial surface of tumor vessels, inhibit VEGFR can significantly suppress liver metastasis of BC. Hypoxia induced factor (HIF)-1 can activate TWIST and promote the binding of VEGF to VEGFR to contribute BCLM (92).

In addition, activated HSCs suppress antitumor immune response by inducing T cell apoptosis and releasing TGF-β (46, 93), consistent with the well-established notion that immune suppression by CAFs is mediated by CXCL12 or nicotinamide adenine dinucleotide phosphate oxidase 4 (NOX4), leading to exclusion of CD8^+^ T cells^85,86.^ Notably, HSCs can modify the extracellular matrix (ECM), thereby facilitating or impairing BC cell migration and invasion (89, 90). Relaxin (RLN), an anti-fibrosis peptide in liver tissue preferentially target metastatic BC cells and activated HSCs. The increased expression of RLN can inhibit BCLM, where RLN gene might be a novel target for treating BCLM (92).

### Neutrophils

Neutrophils are innate immune cells. Clinical studies have demonstrated that increased neutrophil-to-lymphocyte ratio or immune-infl;ammation index is associated with poor survival in BC patients (109–111). Neutrophils can inhibit tumor growth through releasing cytolytic factors and recruiting CD8^+^ cytotoxic T cells or macrophages in the hepatic microenvironment (40, 54, 99). On the other hand, neutrophils have also been shown to promote cancer progression and metastasis *via* distinct mechanisms. For example, they can anchor circulating BC cells and enhance migration of BC cells. The tumor-interacting neutrophils may promote BCLM in a CD90-TIMP-1 juxtacrine-paracrine manner (55). Of note, a recent report showed that aged neutrophils can robustly enhance BCLM through releasing neutrophil extracellular traps, reactive oxygen species, VEGFs, and MMP-9 (96), which are involved in the well-known neutrophil response to infection and injury.

### Myeloid-derived suppressor cells and regulatory T cells

The myeloid-derived suppressor cells (MDSC) and regulatory T cells (Tregs) are known as immunosuppressive cells, and they induce an immune tolerance state that permits tumor growth by evading T-cell-mediated killing (47, 48, 65, 112). MDSCs can be recruited to the liver metastasis site by chemokines released by LSECs, KCs and HSCs (100). These MDSCs, especially S100A8/Gr1-positive MDSCs (101), can enhance the growth and aggressiveness of BC cells and consequently liver metastasis by producing arginase and IL-6 (102–104). Much effort has gone into the development of approaches to eliminate MDSCs, but these attempts have been met with little success (113). Notably, studies have shown that Tregs can exacerbate the development of liver metastasis in intra-abdominal malignancies (114). A direct relationship between Treg accumulation and BCLM has not been reported yet, and there remains an intriguing question as to the role of Tregs in BCLM.

## Prognostic factors of breast cancer liver metastasis

Liver metastatic cancer cells spread through the systemic circulation and therefore liver metastases are rarely isolated (115). Only about 5-10% of patients with BCLM have isolated metastases confined to the liver with no evidence of metastatic disease at other sites (12, 116). The median survival time of untreated patients with BCLM is limited to a few months and is dependent on several prognostic factors (12, 117).

Survival of patients with metastatic BC is affected by many different clinical features, including age, race, marital status, performance status, tumor size, lymph node status, number of metastatic sites, history of treatments, and subtype (118). For BCLM patients, Eichbaum et al. found a prognostic benefit for BCLM patients who were hormone receptor (HR)+ and had an expression of Ki-67 <20% and p53 <50% (119). Similarly, in a review of 4,285 BCLM patients, Xie et al. found that those with the HR+/HER-2+ subtype had the longest median survival of 31.0 months. Patients who were HR-/HER-2+ had a median survival of 22.0 months, and those who had triple negative breast cancer (TNBC) had the shortest median survival of 8.0 months (120). It has been observed that patients with TNBC have the lowest survival after liver metastases in many other studies as well, given the lack of effective therapy (121, 122). In general, factors that may predict worse survival after liver metastasis include the triple negative phenotype, time from curative therapy to liver metastases, burden of tumor cells, and high histological grade of primary (115, 123, 124).

To date, there are few studies on prognostic molecular markers for patients with BCLM. Tian et al. showed that mutations in AKT1, ESR1, ERBB2, FGFR4, APOBEC cytidine deaminase, and defective DNA mismatch repair were significant genetic determinants for BCLM development and progression (125). The study by Yang et al. found that the PPFIA1 gene was markedly elevated in BCLM and associated with decreased disease-free survival (DFS) in HR+ BCLM patients (126). In HER-2+ BC patients, mutant CCND1 (P241P) and PIK3CA (E542K) led to significantly reduced DFS (127).

## Current systemic therapies for breast cancer liver metastasis

Systemic therapy remains the cornerstone of BCLM management. However, BCLM patients who are treated with systemic therapy have poor survival, particularly for triple negative or HR- subtypes (128). If treated with chemotherapy alone, the median survival of BC patients with solely liver metastasis or with limited disease elsewhere is between 19 months (with pre-taxane chemotherapy regimens) to 22-26 months (with taxane-containing regimens) (129). The five-year overall survival (OS) of patients with BCLM treated with systemic therapy is 8-12% (117, 130). It is highly likely that new systemic therapies recently approved for TNBC and HER-2+ BC may improve clinical outcomes in patients with BCLM.

For patients with non-TNBC subtypes, there are options for targeted systemic therapies. In the setting of metastatic HER-2+ tumors, trastuzumab in combination with systemic therapy is associated with longer OS and progression-free survival (PFS) compared to those treated with systemic therapy (14, 131, 132). Rossi et al. found that in patients with metastatic HER-2+ BC who were treated with trastuzumab and had liver-lung metastases (n=328), 4-year survival was 32.1% (133). Further, there has been significant innovation in HER-2-directed therapies in recent years. Currently, there are two Food and Drug Administration (FDA)-approved HER-2-directed antibody-drug conjugates (ADCs), trastuzumane-emtansine (T-DM1) and trastuzumab-deruxtecan (T-DXd), for HER-2+ metastatic BC (134). Interestingly, the DESTINY-Breast04 clinical trial showed that T-DXd also prolonged PFS and OS in HER-2-low metastatic BC patients than chemotherapy (135). Recently, the third HER-2-directed ADC, disitamab vedotin (RC48), received approval for treatment of metastatic gastric or gastroesophageal junction cancer in China in 2021. It may also soon become a treatment modality for HER-2+ metastatic BC (134). At present, there are a total of 11 ADCs that target HER family receptors in clinical trials.

In terms of HR+ disease, endocrine therapy in combination with cyclin-dependent kinase 4 & 6 inhibitors have shown to be effective in patients with both bone-only and visceral metastases (136). He et al. reviewed HR+ patients who were treated with fulvestrant. Fifty-one patients had liver-only metastases, and their PFS was 3.7 months (137). Recently, the SOLAR-1 trial revealed that the alpelisib plus fulvestrant treatment had a significantly benefit in median OS (37.2 months) compared to fulvestrant alone (22.8 months) for PIK3CA-mutant/HR+/HER-2- BC patients with lung and/or liver metastasis (138). Further, several new oral bioavailable selective estrogen receptor modulators/degraders (SERMs/SERDs), including lasfoxifene, bazedoxifene, LSZ102, and RAD1901, for ESR1 gene mutation are currently under clinical investigations to treat ESR1-mutant or endocrine therapy resistant metastatic BC (139). It remains to be determined whether SERMs/SERDs are effective treatment modalities for BCLM.

In contrast to other BC subtypes, there is no first-line targeted therapy for TNBC. The clinical impact of chemotherapy as the standard treatment of TNBC is limited (140). Sacituzumab govitecan (SG) is an antibody-drug conjugate composed of antibody targeting human trophoblast cell-surface antigen 2 (Trop-2), coupled to topoisomerase I inhibitor (SN-38) *via* a proprietary hydrolyzable linker. In ASCENT study, 42% of patients had liver metastasis. The median PFS (5.6 months) and OS (12.1 months) of metastatic TNBC patients in the SG group were significantly longer than in the chemotherapy group (141). Furthermore, immune checkpoint inhibitors (ICIs) in several clinical trials have shown positive results for treating metastatic BC recently (142). A phase III trial (KEYNOTE-355) utilized pembrolizumab plus chemotherapy to treat metastatic TNBC. For patients with PD-L1 expression (combined positive score [CPS] ≥10), median PFS was 9.7 months in pembrolizumab group vs. 5.6 months in placebo group (143). Another study used pembrolizumab and capecitabine for metastatic BC (144). However, metastatic BC only had moderate response to ICIs. PD-L1 positive, first-line therapy, high tumor-infiltrating lymphocytes, and high CD8+ T cell infiltrating are associated with better response to ICIs (142). BCLM reportedly had a lower response rate to ICI treatment when compared with the other metastatic locations (142). Therefore, exploring new targets and developing more effective therapy for BC with liver metastasis are urgently needed and would have tremendous clinical impact.

## Hepatic microenvironment related therapeutic implications for BCLM

Given the critical roles of different cells in the hepatic microenvironment in BCLM formation, it is tempting to explore novel therapeutic approaches based on available interventional agents targeting the key signaling proteins in these cells. These potential treatment options for BCLM are summarized in **Table 3** and described below.

**Table 3:** Hepatic microenvironment related therapeutic implications in breast cancer liver metastasis.

Hepatic micoenvironment cells	Related moleculars or pathway	Therapeutic implications	References
Breast cancer stem cells	CD44, TGF-β1 pathway	bivatuzumab mertansine	(68, 69, 145)
Hepatocytes	Claudin-2, ECM components (such as fibronectin and type IV collagen), integrin complexes	Lyn-selective kinase inhibitor Bafetinib (INNO-406)	(52, 79–81)
	E-cadherin, ERK pathway	ROS1 inhibitors (crizotinib)	(82, 146, 147)
Macrophages	STAT6, IL-4 and IL-13, CD47	PLD inhibitors [FIPI (dual PLD1/PLD2 inhibitor) or VU0155072-2 (PLD2 inhibitor)], cabazitaxel	(58, 86, 148, 149)
NK cells	Interleukin-15, interferon-γ, CXCL12 and CXCR4	Interleukin-15 based immunotherapy	(94)
Neutrophils	G-CSF, P53, WNT, KIAA1199, TGFβ-CXCL3/1-CXCR2 axis	LGK974 (a Porcupine inhibitor blocking acylation of Wnt), KIAA1199 inhibitors	(97, 98, 150–152)

ECM, Extracellular matrix; PLD, Phospholipase D; NK cells, Natural killer cells; TGF-β, Transforming growth factor-β; ERK, Extracellular regulated protein kinases; IL, Interleukin; STAT, Signal transducer and activator of transcription; CXCL, Chemokine (C-X-C Motif) ligand; CXCR, Chemokine (C-X-C motif) receptor; G-CSF, Granulocyte colony stimulating factor. Figure legends.

For BC stem cells in the hepatic microenvironment, the cell surface adhesion molecule CD44 has been shown to potentiate the invasion and metastasis of BC cells to the liver (68, 69). These studies suggest that CD44 may be a novel target for inhibiting BCLM. One phase I study utilized bivatuzumab mertansiene to treat metastatic BC patients with positive CD44v6, and estimated the pharmacokinetics and safety of the treatment (145). This study demonstrated that the bivatuzumab mertansine targeting CD44v6 could be a potential therapeutic option for metastatic BC patients that express CD44v6 (145).

Claudin-2 is a molecule that plays a key role in the formation of tight junctions. Previous studies have shown that liver metastatic BC cells express high levels of Claudin-2 and the protein is critical for the adhesion between BC cells and hepatocytes by acting as an adhesion molecule (52). Afadin, a Claudin-2-interacting partner, is also involved in BC cell metastasis to the liver (79). Furthermore, the Claudin-2 expressed by liver metastatic BC cells can increase the expression of integrin complexes in the surface of BC cells, leading to enhanced adhesion of BC cells to ECM components, such as type IV collagen and fibronectin (80). Although primary human BC samples express low levels of Claudin-2, most of the liver metastatic BC samples were found to display higher expression levels of Claudin-2 (80). Further supporting the significant role of Claudin-2 in BCLM formation, Tabaries et al. demonstrated that blocking tumor-hepatocyte interactions by inhibiting Claudin-2 expression using the Lyn-selective kinase inhibitor Bafetinib (INNO-406) can suppress BCLM growth (81). Currently, most studies on Bafetinib are in the preclinical stage, and there are a few phase I and phase II clinical trials of Befetinib in chronic leukemia, prostate cancer, and brain cancer.

The epithelial cell adhesion E-cadherin may also play an important role in BC cell interaction with hepatocytes. BC cells, which undergo EMT to escape from primary tumors, have been shown to re-express E-cadherin upon entering the liver microenvironment (82). This upregulation contributes to the adhesion with hepatocytes and promotes BC cell survival by activating extracellular regulated protein kinases (ERK) signaling (82, 146). Although the clinical relevance of breast tumor-hepatocyte or breast tumor-ECM interactions has not been well evaluated, disruption of interactions between BC cells and hepatocytes or ECM may serve as a potential strategy to inhibit BCLM. Furthermore, results of some preclinical studies suggest that ROS1 inhibitors, such as crizotinib, may be utilized to treat E-cadherin defective BC. The preclinical data provided theoretical basis to support the phase II clinical trials to evaluate the safety and efficacy of ROS1 inhibitors in E-cadherin defective BC patients (147). Currently, ROS1 inhibitors are widely used to treat lung cancer patients with ROS1 mutations, and there are also phase I and phase II clinical trials for ROS1 inhibitors to treat patients with other advanced or metastatic solid tumors.

For macrophages, targeting M2 macrophage polarization has been proposed as an anti-cancer treatment approach. The STAT6, a key effector and mediator of IL-4 and IL-13 function, is a potential therapeutic target in this regard (58). Notably, phospholipase D-2 (PLD-2) is an important player in BC progression and metastasis. In preclinical studies, PLD inhibitors [FIPI (dual PLD1/PLD2 inhibitor) or VU0155072-2 (PLD2 inhibitor)] were found to reduce the tumor-promoting macrophages and neutrophil infiltration in primary breast tumors and liver metastasis, thereby suppressing BCLM (86). In addition, Cao et al. showed that cabazitaxel could affect macrophages and improve the immunotherapy targeting CD47 in TNBC (148). The activation of macrophages by cabazitaxel combined with the CD47 blocking effect could drastically enhance the tumoricidal activity against TNBC cells, thus suppressing BCLM (148). Cabazitaxel is widely utilized to treat patients with metastatic castration resistant prostate cancer. Several phase I and phase II trial studies have shown the efficacy of cabazitaxel in metastatic BC patients so far (149). One recently study showed an increased NK cells in dormant heptic microenvironment. Interleukin-15 based immunotherapy could ensure a large number of NK cells to maintain dormancy and prevent BCLM through interferon-γ signalling (94). Actived HSCs secreted chemokine CXCL12 could induce NK cells quiescence through CXCR4 to promote BCLM (94). Normalizing NK cell pool might be a novel way to prevent BCLM.

For neutrophils, the immature low-density neutrophils (iLDNs) mobilized by cancer cell-derived granulocyte colony stimulating factor (G-CSF) can promote BCLM (150), and the liver metastatic growth may be facilitated by neutrophil-derived transferrin in BC (97). The neutrophil or the transferrin receptors depletion could inhibit transferrin production in the metastatic microenvironment and suppress BC metastasis (97). Furthermore, P53 may be a key regulator of pro-metastatic neutrophils. It was shown that blockade of Wnt secretion by LGK974, a Porcupine inhibitor blocking acylation of Wnt, or shRNA in p53-null BC cells reverse subsequent neutrophilic inflammation, resulting in reduced BCLM growth (98). To date, the WNT inhibitor LGK974 is under a phase I clinical study in patients with malignancies (151). Recently, Wang et al. found that the KIAA1199 could promote immunosuppressive neutrophils to infiltrate into the liver microenvironment, which indicated that KIAA1199 might be a potential therapeutic target to treat the liver metastasis (152). In addition, KIAA1199 carries out its function through the TGFβ-CXCL3/1-CXCR2 signaling pathway. Restoration of immune infiltration in the liver metastasis microenvironment can potentially be achieved by inhibiting KIAA1199 pharmacologically, and thereby allowing for suppression of liver metastasis and enhancement the response to ICI treatment (152). Further *in vivo* studies and clinical trials are needed to establish the utility of KIAA1199 inhibition in the treatment of BC with liver metastasis.

## Conclusion

Although recent advances in the understanding of the molecular mechanisms of hepatic microenvironment in BCLM using various *in vitro* and *in vivo* models shed light on potential therapeutic targets, much work is needed to tie these findings to clinical relevance. Concomitantly, identification of biomarkers to predict BCLM risk, progression, treatment response, and patient survival will have a significant clinical impact. Further in-depth investigation of critical pathways and genetic changes underlying human BCLM would pave the way for developing new approaches for preventing and treating BCLM.

## Author contributions

All authors listed have made a substantial, direct, and intellectual contribution to the work and approved it for publication.

## Funding

XC is supported by National Institutes of Health (2R01CA151610), Department of Defense (W81XWH-18-1-0067) and Samuel Oschin Cancer Institute research development fund. AG is supported by the Fashion Footwear Charitable Foundation of New York, Inc., the Margie and Robert E. Petersen Foundation, and Linda and Jim Lippman Fund. This study is also supported by the National Natural Science Foundation of China (No. 81602189) and CSCO-Genecast Cancer Precision Therapy Research Fund (Y-2019Genecast-039). The funder was not involved in the study design, collection, analysis, interpretation of data, the writing of this article or the decision to submit it for publication.

## Conflict of interest

Author RB has a consulting role for Navartis, Biotheranostics, AstraZeneca, Seattle Genetics and Gilead Inc. She is the honoraria of MJH Healthcare, WebMD, and AstraZeneca, and also a speaker for Eli Lilly. She is supported by the research fundings from Merck, Seattle Genetics, Takeda, Pfizer, and Eli Lilly.

The remaining authors declare that the research was conducted in the absence of any commercial or financial relationships that could be construed as a potential conflict of interest.

## Publisher’s note

All claims expressed in this article are solely those of the authors and do not necessarily represent those of their affiliated organizations, or those of the publisher, the editors and the reviewers. Any product that may be evaluated in this article, or claim that may be made by its manufacturer, is not guaranteed or endorsed by the publisher.
